# Renoprotective Effects of Aldose Reductase Inhibitor Epalrestat against High Glucose-Induced Cellular Injury

**DOI:** 10.1155/2017/5903105

**Published:** 2017-03-12

**Authors:** Heba El Gamal, Ali Hussein Eid, Shankar Munusamy

**Affiliations:** ^1^College of Pharmacy, Qatar University, P.O. Box 2713, Doha, Qatar; ^2^Department of Pharmacology and Toxicology, School of Medicine, American University of Beirut, Beirut, Lebanon

## Abstract

Diabetic nephropathy (DN) is the leading cause of end stage renal disease worldwide. Increased glucose flux into the aldose reductase (AR) pathway during diabetes was reported to exert deleterious effects on the kidney. The objective of this study was to investigate the renoprotective effects of AR inhibition in high glucose milieu in vitro. Rat renal tubular (NRK-52E) cells were exposed to high glucose (30 mM) or normal glucose (5 mM) media for 24 to 48 hours with or without the AR inhibitor epalrestat (1 *μ*M) and assessed for changes in Akt and ERK1/2 signaling, AR expression (using western blotting), and alterations in mitochondrial membrane potential (using JC-1 staining), cell viability (using MTT assay), and cell cycle. Exposure of NRK-52E cells to high glucose media caused acute activation of Akt and ERK pathways and depolarization of mitochondrial membrane at 24 hours. Prolonged high glucose exposure (for 48 hours) induced AR expression and* G*1 cell cycle arrest and decreased cell viability (84% compared to control) in NRK-52E cells. Coincubation of cells with epalrestat prevented the signaling changes and renal cell injury induced by high glucose. Thus, AR inhibition represents a potential therapeutic strategy to prevent DN.

## 1. Introduction

Diabetic nephropathy (DN) is the leading cause of end stage renal disease worldwide [1]. Chronic hyperglycemia causes progressive kidney damage in diabetic patients, which is characterized by three main findings: (1) persistent albuminuria (more than 300 mg/day or more than 200 *µ*g/min), (2) progressive and irreversible decline in glomerular filtration rate (GFR), and (3) increased arterial blood pressure [2]. In most DN cases, pathological changes are prominent in the glomeruli as well as the renal tubules of diabetes patients' kidneys even before the onset of microalbuminuria. In the glomeruli, the early adaptive changes are marked by glomerular hypertrophy and thickening of the glomerular basement membrane (GBM). As the disease progresses, the mesangial matrix expands, and the mesangial cells increase in size, leading to glomerulosclerosis and the development of nodular lesions also known as Kimmelstiel-Wilson nodules, a hallmark of DN. Similarly, the tubules also undergo early hypertrophy and thickening of the tubular basement membrane (TBM), which then progress to tubulointerstitial fibrosis and inflammation at later stages of the disease [3].

Growing body of evidences has shown that DN is induced by multiple factors such as impaired glucose signaling, increased blood pressure, imbalance in the cellular redox status, inflammation and altered protein folding, and turnover within the renal cells. Additionally, several signaling pathways are implicated in the development and progression of DN [4]. Akt, a 57 KDa serine/threonine kinase that regulates numerous cellular functions such as transcription, translation, cell proliferation, and survival, is thought to play a critical role in induced complications [5]. It has been shown that Akt increases the production of extracellular matrix (ECM) proteins such as collagen-I, collagen-IV, and laminin in diabetic kidney [6, 7]. In addition, the activation of PI3K/Akt pathway in renal tubules has been shown to cause tubular hypertrophy via two mechanisms: (1) by a cell cycle-dependent mechanism and (2) by altering protein translation through phosphorylation of the protein translation regulator 4E-BP1 [8–10].

The extracellular signal-regulated kinase (ERK) pathway is another crucial signaling network involved in numerous cellular processes [11]. Under hyperglycemia, the Ras-Raf-MEK-ERK signal transduction cascade becomes activated and leads to the activation of renin-angiotensin-aldosterone-system (RAAS), which is well known to cause renal injury [4, 12]. Moreover, ERK has been shown to induce vascular endothelial growth factor (VEGF) and transforming growth factor-beta 1 (TGF-*β*1), which causes albuminuria, increase in GFR, and kidney fibrosis [4, 10, 13]. Furthermore, several studies have showed crosstalk between ERK pathway and other pathways in DN such as advanced glycation end products (AGE) and protein kinase C (PKC) pathways [14]. Therefore, ERK activation is considered one of the significant signaling events in DN.

Aldose reductase (AR; EC 1.1.1.21) is a member of aldo-keto reductase (AKR) superfamily [15], which constitutes more than 100 structurally related proteins [16]. AR is the rate-limiting enzyme in the polyol pathway, which is responsible for the reduction of glucose to sorbitol using NADPH as a cofactor. Subsequently, sorbitol is converted to fructose by sorbitol dehydrogenase using NAD+ as a cofactor. In normoglycemic conditions, glucose serves as a poor substrate for AR due to the high Km values of AR for glucose. Under hyperglycemic conditions, there is increase in flux of glucose through polyol pathway, which leads to imbalances in cofactors and products causing several deleterious effects such as osmotic stress, oxidative stress, increased AGE, and activation of PKC pathway [17].

Many studies have investigated the role of polyol pathway in the development of DN and evaluated the protective role of various aldose reductase inhibitors (ARIs) [18–24]. ARIs are thought to exert their beneficial effects by decreasing hyperfiltration, albuminuria, and accumulation of ECM proteins (antifibrotic effects) without affecting the blood glucose or HbA1c levels [25]. In addition, plethora of in vitro data supports the link between polyol pathway and PKC activity and TGF-*β*1 and ECM protein production, which suggest the involvement of this pathway in fibrotic changes observed in DN [26–29]. Although several preclinical studies using ARIs have demonstrated nephroprotective effects against DN, the results from most clinical trials are not very encouraging [25]. There are several challenges to be faced when targeting AR enzyme including the difficulty of developing ARIs with high specificity towards ALR2 isoform and the use of optimum doses for AR inhibition. Moreover, the efficacy of ARIs depends on the levels of expression of AR in target tissues and the degree of involvement of polyol pathway in the development of diabetic complications, which varies significantly across diabetic patients [25, 30].

In this study, we have investigated the effects of high glucose concentrations on rat renal proximal tubular cells cultured in vitro. The study is aimed to investigate the key molecular pathways that are activated in renal cells under high glucose conditions and to identify the cellular markers that mediate hyperglycemia-induced renal cell injury. We hypothesized that exposing renal tubular cells to high glucose concentrations would induce AR expression and, thus, inhibiting AR using epalrestat (a potent ARI) would confer protection against high glucose-induced renal cell injury.

## 2. Materials and Methods

### 2.1. Cell Culture

Normal rat kidney cells (NRK-52E; Health Protection Agency, UK) were cultured in Dulbecco's modified Eagle medium (DMEM, Life Technologies, UK) supplemented with 10% fetal bovine serum (FBS), 1% L-glutamine, and 1% penicillin/streptomycin. The cells were maintained at 37°C in 5% CO_2_ incubator. To obtain an in vitro model that represents the features of DN, cells were divided into 3 groups: (1) control, where cells were treated with DMEM medium containing 5 mM glucose and 1% FBS, (2) high glucose treated, where cells were treated with DMEM medium containing 30 mM glucose and 1% FBS, and (3) osmotic control, where cells were treated with DMEM medium containing 5 mM glucose and 25 mM mannitol with 1% FBS to simulate a milieu that is osmotically equivalent to the 30 mM high glucose media. The cells were exposed to the above-mentioned conditions for 24, 48, and 72 hours. To investigate the effects of AR inhibition on high glucose-induced stress in renal cells, NRK-52E cells were cotreated with epalrestat (Sigma Aldrich, Germany) at 1 *µ*M concentration and compared against vehicle-treated controls.

### 2.2. Assessment of Cell Viability

NRK-52E cells were seeded on 96-well plates and treated according to the aforementioned protocol. At the end point, media were removed and replaced by 100 *µ*l of fresh serum-free media and 10 *µ*l of 5 mg/ml MTT (3-(4,5-dimethylthiazol-2-yl)-2,5-diphenyltetrazolium bromide) reagent (Sigma Aldrich, Germany). The plates were incubated at 37°C for 3-4 hours. The purple formazan crystals formed were solubilized in 50 *µ*l of dimethylsulfoxide (DMSO), and the absorbance was measured at 570 nm using a microplate reader. Absorbance value from the control group was set as 100% and the values from treatment groups were expressed as percentage of control.

### 2.3. Western Blotting

NRK-52E cells were seeded on 6-well plates. At the end point, cells were scrapped and lysed in 60 mM Tris, pH 6.8 buffer containing 2% SDS. Samples were sonicated for 10–15 seconds and centrifuged at 16,000 ×g for 15 minutes at 4°C prior to determination of protein concentrations using bicinchoninic acid (BCA) protein assay (Pierce, USA). About 20 to 40 *µ*g of protein from each group was loaded onto 10% or 12% sodium dodecyl sulfate-polyacrylamide gels (SDS-PAGE) and resolved by electrophoresis and transferred onto polyvinylidene fluoride (PVDF) membranes. The membranes were blocked with 5% nonfat milk for 1 hour and then probed with specific primary antibodies (overnight at 4°C) for the following: aldose reductase (ab131182 Abcam, 1 : 1000, mouse), p-Akt (ab81283 Abcam, 1 : 2000, rabbit), Akt (ab32505 Abcam, 1 : 2000, rabbit), p-ERK (9106 cell signaling, 1 : 1000, mouse), and ERK (9102 cell signaling, 1 : 1000, rabbit). Subsequently, membranes were incubated with horseradish peroxidase- (HRP-) conjugated goat anti-mouse IgG (1 : 10,000; Abcam, UK) or goat anti-rabbit IgG (1 : 20,000; Abcam, UK) secondary antibodies for 1 hour at room temperature. The immunoreactivity was visualized with Optiblot ECL detection kit (Abcam, UK) using Syngene (G-BOX Chemi XX6) imaging system and analyzed using Scion Image software. The densitometry values were normalized to actin (used as loading control) and expressed as percentage of control.

### 2.4. Assessment of Mitochondrial Membrane Potential (JC-1 Assay)

The high glucose-induced alterations in the mitochondrial membrane potential in NRK-52E cells were assessed using JC-1 (5,5′,6,6′-tetrachloro-1,1′,3,3′ tetraethylbenzimidazolylcarbocyanine iodide; T-3168, Life Technologies, UK) as described previously [31]. Briefly, after 24 or 48 h high glucose treatment, cells were stained with JC-1 reagent (10 *µ*M) for 30 minutes at 37°C in dark, and the fluorescence was measured using a microplate reader (SpectraMax, USA) at excitation/emission (Ex/Em) 488 nm/529 nm to measure monomers and Ex/Em 488 nm/590 nm to measure J-aggregates. The ratio of fluorescent intensity of J-aggregates (red) to that of monomers (green) was calculated to determine changes in mitochondrial membrane potential. For fluorescence microscopy, cells were stained with JC-1 as described above and imaged under fluorescent microscope (Olympus, USA) with TRITC filter at 200x magnification.

### 2.5. Cell Cycle Analysis

Following 24 or 48 h high glucose treatment, NRK-52E cells were fixed in 70% ethanol and kept at −20°C. On the day of analysis, cells were pelleted out by centrifugation at 1000 ×g for 5 minutes at 4°C and stained with Tali® cell cycle solution (Life Technologies, UK) in dark for 30 minutes to achieve a cell density of 1 × 10^5^–5 × 10^6^ cells/ml for analysis using Tali image-based cytometer (Life Technologies, UK).

### 2.6. Statistical Analysis

All values were calculated as a percentage of control (5 mM glucose-treated group) and expressed as Mean ± SEM. One-way ANOVA test followed by Tukey's post hoc test was performed using GraphPad Prism 6 to detect statistical differences between groups. *P* < 0.05 was considered statistically significant.

## 3. Results

### 3.1. Effect of High Glucose on the Viability and Relative Mitochondrial Membrane Potential of Renal Proximal Tubular Cells

**Figure 1:**
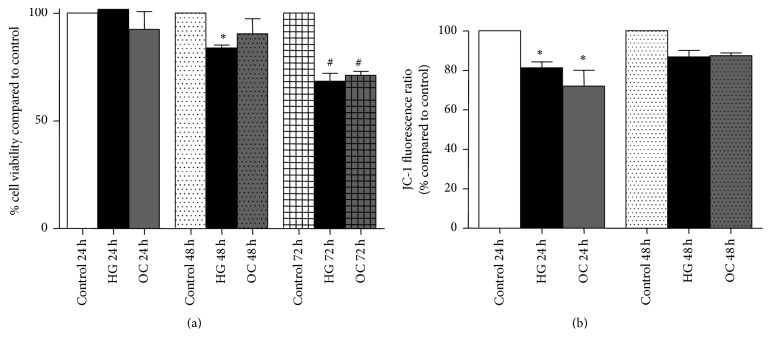
Effect of high glucose (30 mM) on NRK-52E cells viability and relative mitochondrial membrane potential. (a) Cell viability of low passage number cells (P2–P6) after 24 h, 48 h, and 72 h exposure to high glucose as determined by MTT assay. Absorbance values were normalized to control group and expressed as percentage Mean ± SEM (*n* = 4–7). _ _^*∗*^*P* < 0.05 compared to control 48 h. _ _^#^*P* < 0.05 compared to control 72 h. (b) Effect of high glucose concentration on relative mitochondrial membrane potential in NRK-52E cells after 24 and 48 h exposure using JC-1 staining. Ratios obtained from fluorescence values of J-aggregates (excitation/emission 488/529 nm) and that of monomers (excitation/emission 488/590 nm) were expressed as percentage Mean ± SEM (*n* = 3) compared to control. _ _^*∗*^*P* < 0.05 compared to control 24 h.

NRK-52E cells were exposed to high glucose media (30 mM) for 24, 48, and 72 hours. 30 mM mannitol was used as osmotic control. At the endpoint, cell viability was assessed by MTT assay. It was observed that when using cells at low passage number (P2 to P6), there was a significant decrease in viability after 48 hours to 84%, which further declined to 68% after 72 h (Figure 1(a)). Intriguingly, the high glucose-induced decrease in viability after 48 and 72 h exposure was not reproducible when using cells with passage numbers higher than P7 (data not shown). Thus, we chose to perform all experiments on NRK-52E cells between passage numbers P2 to P6.

Alterations in the relative mitochondrial membrane potential (Δ*ψ*m) of NRK-52E cells following exposure to high glucose concentrations were assessed using JC-1 staining. JC-1 is a cationic dye, which at high Δ*ψ*m forms J-aggregate with red fluorescence, while at low Δ*ψ*m it remains as monomers with green fluorescence. Therefore, the ratio of red to green fluorescence is indicative of the polarization status of the mitochondria membrane. NRK-52E cells exposed to 30 mM glucose for 24 h and stained with JC-1 showed increased green fluorescence, and the red to green fluorescence ratio was 81% as compared to control. Although the mitochondrial depolarization was sustained after 48 h of high glucose exposure, it was not statistically significant (Figure 1(b)). These findings indicate that acute high glucose exposure induces transient mitochondrial depolarization in renal tubular cells.

### 3.2. Effect of High Glucose on Akt and ERK Signaling Pathways in Renal Tubular Cells

**Figure 2:**
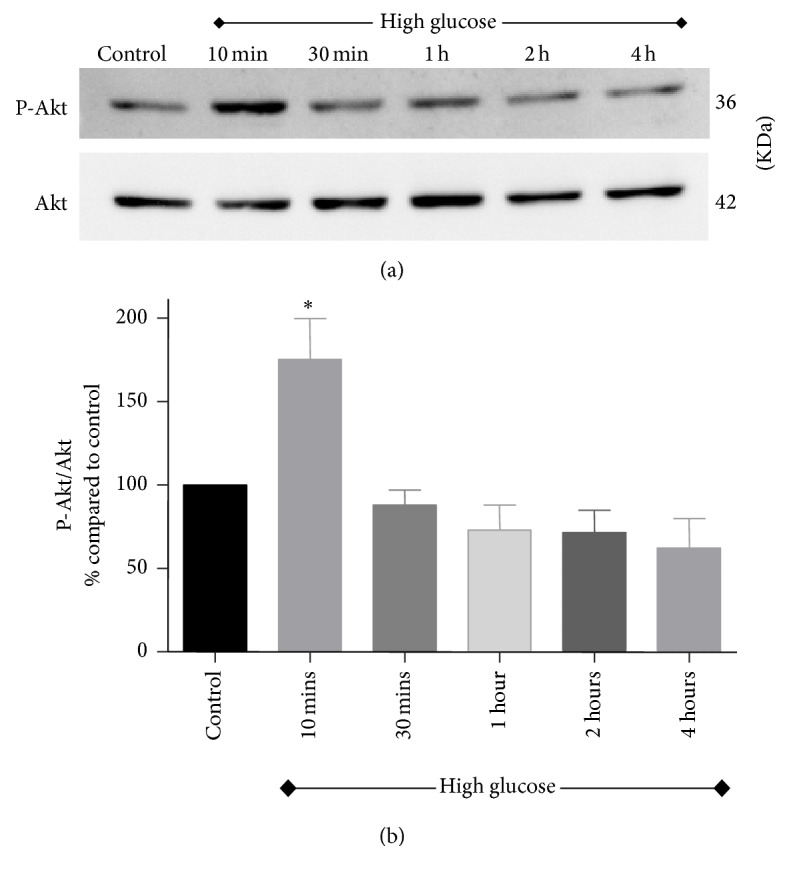
Activation of Akt pathway in NRK-52E cells following exposure to high glucose for 10 minutes, 30 minutes, 1 hour, 2 hours, and 4 hours. (a) A representative western blot showing the expression of p-Akt and total Akt. (b) Bar graph showing densitometry data of p-Akt expression normalized to total Akt expression. Values were expressed as percentage Mean ± SEM (*n* = 3) relative to control. _ _^*∗*^*P* < 0.05 compared to control.

**Figure 3:**
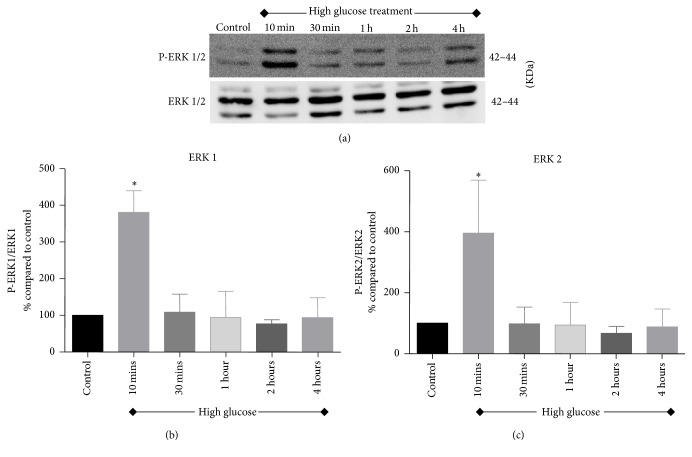
Activation of ERK signaling pathway assessed after exposing NRK-52E cells to high glucose medium for 10 minutes, 30 minutes, 1 hour, 2 hours, and 4 hours. (a) Representative western blots of p-ERK1/2 and ERK1/2. (b) Densitometric analysis for p-ERK1 and (c) densitometric analysis for p-ERK2. Values were expressed as percentage Mean ± SEM (*n* = 3) relative to control. _ _^*∗*^*P* < 0.05 compared to control.

Activation of Akt and ERK signaling pathways was investigated in renal tubular cells following exposure to high glucose for 10 minutes, 30 minutes, 1 hour, 2 hours, and 4 hours. As shown in Figures 2 and 3, there was an acute and transient induction of p-Akt and p-ERK after 10 minutes of high glucose exposure, which then subsided at further time points tested.

### 3.3. Effect of High Glucose on Aldose Reductase (AR) Expression in Renal Tubular Cells

**Figure 4:**
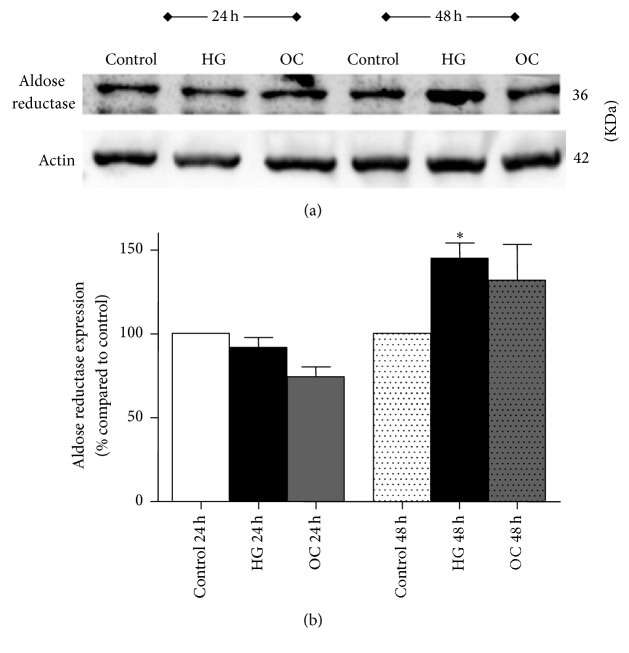
Effect of high glucose (30 mM) on aldose reductase (AR) expression in NRK-52E cells after 24 and 48 h as measured by western blotting. (a) A representative western blot showing the expression of AR in comparison to the loading control *β*-actin. (b) Bar graph with densitometric analysis of AR expression (normalized to *β*-actin). Values were expressed as percentage Mean ± SEM (*n* = 3) relative to control. ^*∗*^*P* < 0.05 compared to control.

To investigate if aldose reductase (AR) was induced in the model, AR expression was measured in NRK-52E cells subjected to high glucose treatment. Although the AR expression remained unchanged after 24 hours of high glucose treatment, it was significantly induced at 48 hours by about 1.5-fold as compared to control (Figure 4). A small induction in AR protein expression was also observed in cells exposed to high osmolar media (30 mM mannitol) at 48 hours.

### 3.4. Effect of Epalrestat on High Glucose-Induced Alterations in Renal Cell Viability and Cell Cycle

**Figure 5:**
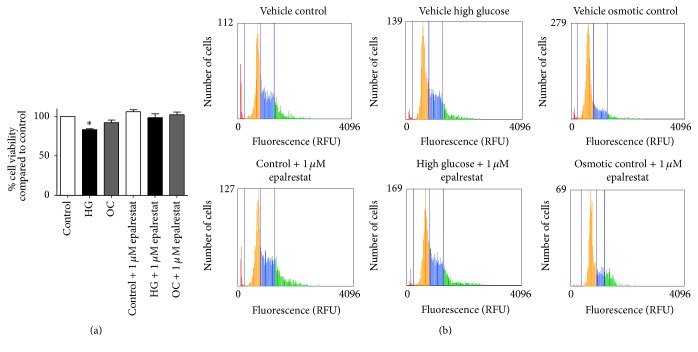
Effects of epalrestat on high glucose-induced renal cell injury and cell cycle progression. (a) Cell viability of NRK-52E cells after exposure to high glucose with and without 1 *µ*M epalrestat for 48 h as determined by MTT assay. Values were expressed as percentage Mean ± SEM (*n* = 3) relative to vehicle-treated control. _ _^*∗*^*P* < 0.05 compared to control. (b) Distribution of NRK-52E cells along cell cycle after incubation with high glucose and mannitol in the presence or absence of epalrestat (1 *μ*M). Summarized data were reported in Table 1.

To examine the protective effect of aldose reductase (AR) inhibition on high glucose-induced renal cell injury, NRK-52E cells were exposed to high glucose in presence of 1 *µ*M epalrestat (an AR inhibitor) for 48 hours. At the endpoint, cell viability was measured using MTT assay. As observed earlier, with 48 hours' high glucose treatment, the cell viability declined to 84% (*P* < 0.05) to that of control. In contrast, coincubation of cells with epalrestat at 1 *µ*M was able to attenuate high glucose induced cell death (Figure 5(a)). In addition, when cells were exposed to high glucose medium for 48 h in presence of epalrestat, the percentage of cells increased in the G1 phase when compared to control but was not statistically significant (Figure 5(b)).

### 3.5. Effect of Epalrestat on Mitochondrial Membrane Potential Depolarization in Renal Tubular Cells

**Figure 6:**
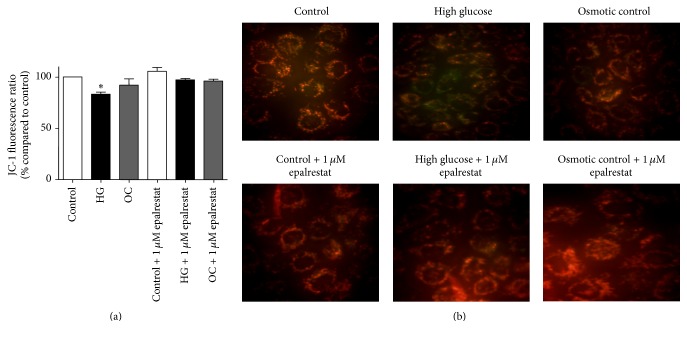
Assessment of relative mitochondrial membrane potential in NRK-52E cells by JC-1 staining. (a) Bar graph showing fluorescence values after 24 h of high glucose exposure with and without 1 *µ*M epalrestat using JC-1 staining. Values were expressed as percentage Mean ± SEM of J-aggregate (excitation/emission 488/529) to monomer (excitation/emission 488/590) ratio compared to control. _ _^*∗*^*P* < 0.05 compared to control. (b) Representative JC-1 stained images of NRK-52E cells treated with high glucose and osmotic control media in the presence or absence of 1 *µ*M epalrestat visualized under 400x magnification using fluorescence microscope.

The effect of epalrestat on the mitochondrial membrane potential in renal tubular cells was assessed after 24 hours of high glucose exposure using JC-1 staining. Coincubation of cells with 1 *µ*M epalrestat reversed the depolarization observed with 24 hours' high glucose treatment. Fluorescent cell images further confirmed this finding, that is, an intense green staining observed in high glucose-treated cells, whereas the cells coincubated with epalrestat show a mixed red and green staining pattern similar to control cells (Figure 6).

### 3.6. Effect of Epalrestat on High Glucose-Induced Activation of Akt and ERK Pathways in Renal Tubular Cells

**Figure 7:**
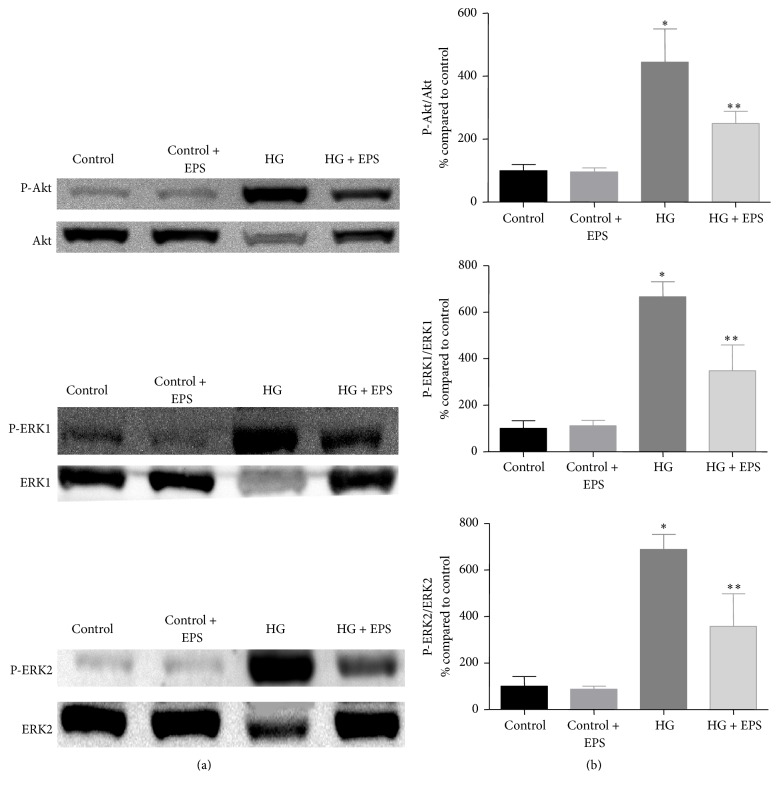
NRK-52E cells were exposed to high glucose (HG) with and without epalrestat (EPS) for 10 minutes and assessed for its effect on Akt and ERK pathways. Left panel showing representative blots for Akt and ERK1 and ERK2, and the right panel showing densitometric analysis of the bands. Values were expressed as Mean ± SEM (*n* = 3). _ _^*∗*^*P* < 0.05 compared to control; _ _^*∗∗*^*P* < 0.05 compared to HG-treated group.

NRK-52E cells were exposed to 10 minutes of high glucose in presence of epalrestat to investigate the effect of aldose reductase inhibition on high glucose-induced activation of Akt and ERK pathways. As seen in Figure 7, epalrestat cotreatment attenuated high glucose-mediated induction of Akt and ERK pathways, which suggests crosstalk between these two pathways and the polyol pathway in renal cells under high glucose conditions.

## 4. Discussion

Our studies indicate that exposure of NRK-52E cells to 30 mM glucose causes a progressive decline in cell viability, that is, 84% viability after 48 hours and 68% viability after 72 hours as compared to control (100%). Similarly, a study by Park et al. using primary rabbit renal proximal tubular cells observed inhibition of cell proliferation over 24, 48, and 72 hours following exposure to 25 mM glucose [32]. It has been shown that high glucose induces mitochondrial alterations and dysfunction, which ultimately leads to apoptosis. In the current study, the effect of high glucose on mitochondrial membrane potential was tested in NRK-52E cells using JC-1 dye. After 24 hours, there was a slight decrease (81% compared to control) in mitochondrial membrane potential, which was sustained at 48 h (86% compared to control) indicating that high glucose treatment induces mitochondrial membrane depolarization in renal tubular cells. Our finding is in line with other studies, which demonstrated the onset of mitochondrial dysfunction in human kidney cells (HK-2) following exposure to high glucose medium [33–35]. Therefore, these findings suggest that tubular cell death observed in the current model might be initiated by alterations in mitochondrial function.

PI3K/Akt pathway is a vital pathway that is implicated in numerous cellular processes [36]. Several studies have revealed impairments in the PI3K/Akt pathway during diabetes and signified its role in the development of diabetic complications [8, 37, 38]. For example, hyperglycemia has been shown to induce collagen-I synthesis in mesangial cells by stimulating cytokines such as TGF-*β* and epidermal growth factor (EGF) through PI3K/Akt dependent pathway [37, 39].

In this study, exposing NRK-52E cells to 30 mM glucose caused acute activation of S473-phosphorylated Akt after 10 minutes of exposure, which then subsided at higher time points (30 minutes, 1 h, 2 h, and 4 h). This finding was consistent with other findings previously described in the literature where high glucose activates Akt pathway in renal cells [9, 40].

ERK1/2 pathway is a major pathway that is actively involved in regulation of cellular functions. It has been shown that mitogen-activated protein kinase (MAPK) cascade was activated in glomerular mesangial cells exposed to high glucose concentrations as well as in diabetic rats' glomeruli [41]. Furthermore, inhibiting ERK 1/2 pathway in mesangial cells in vitro attenuated high glucose-induced TGF-*β*1 expression and the induction of collagen-I and fibronectin mRNA [42, 43]. In addition, in vitro studies using renal tubular cells showed that ERK1/2 pathway is implicated in high glucose-induced TGF-*β*1, fibronectin, and collagen-IV expression as well as tubular cell hypertrophy [44, 45]. In the current study, high glucose treatment induced acute phosphorylation of ERK1/2 within 10 minutes of exposure in renal tubular cells. Taken together, these findings provide evidence for the role of ERK1/2 pathway in the development and progression of DN.

Findings from this study indicate that exposing tubular cells to high glucose concentrations in vitro induces low levels of cellular stress and injury, which is not unexpected given the complexity of mechanisms involved in diabetic complications and the inherent limitations of in vitro high glucose model to mimic diabetic setting. To establish an in vitro model of DN within a reasonable time frame, culturing cells at high glucose concentrations are commonly employed [32, 46]. Alternatively, cells could be transfected with markers of DN such as angiotensin II and TGF-*β*1 to aggravate DN in vitro. Additionally, albumin can be used in conjunction with high glucose to mimic advanced stages of DN [47]. Serum (FBS) concentration is another crucial factor in the development of an in vitro DN model. In our model, serum deprivation has greatly affected the growth and morphology of NRK-52E cells, and consistent results were obtained when cells were exposed to 30 mM glucose media containing 1% FBS for 24 and 48 hours.

It has been shown that activation of polyol pathway due to hyperglycemia in renal cells plays a pivotal role in the development of DN [48]. Therefore, it is hypothesized that inhibiting aldose reductase enzyme, the rate-limiting enzyme in the polyol pathway, can protect the cells against high glucose-induced cell injury in renal tubular cells [49]. Activation of polyol pathway in response to high glucose has been reported to cause a sevenfold increase in sorbitol production in renal proximal tubules [50]. Our results reveal that aldose reductase expression was increased by about 1.5-fold with high glucose treatment as compared to control at 48 hours. Similar inductions of AR protein expression have been reported in studies using mesangial cells cultured under high glucose conditions [29, 48].

Coincubation of NRK-52E cells with 1 *µ*M epalrestat (dose based on the literature [34] and our pilot studies) has prevented mitochondrial depolarization and loss of cell viability induced by high glucose. These findings suggest that AR inhibition can protect renal cells against high glucose-induced mitochondrial alterations and improve cell survival. In addition, cell cycle analysis was performed to determine the effects of high glucose on cell cycle progression. Our results showed that incubation with high glucose for 48 h increased the proportion of cells in G1 phase as compared to control (59% versus 51%, resp.). Consistent with our findings, a similar G1 phase arrest has been observed in other studies using different types of renal tubular cells [8]. Together, these findings suggest that tubular renal cell hypertrophy occurs in cell cycle-dependent manner [8, 32, 51].

Although treatment with epalrestat did not block the effects of high glucose on cell cycle in our model, it ameliorated high glucose-mediated acute activation of Akt and ERK pathways. As mentioned earlier, both pathways are actively involved in DN and contribute to development of many of its pathologic features. Therefore, our results suggest that AR inhibition exerts a protective effect on kidney cells through attenuation of Akt and ERK dependent pathways. This finding is in line with another study, which showed that transfecting mesangial cells with AR gene augmented high glucose-mediated activation of Akt and ERK pathways in these cells [29].

In support to our findings, inhibiting aldose reductase has been shown to reverse high glucose-induced production of ECM proteins such as fibronectin [29]. Nevertheless, specific inhibition of aldose reductase with enzyme inhibitors is extremely challenging due to its extensive homology with other aldo-keto reductase family members and makes it hard to find specific inhibitors that are devoid of properties to block other aldo-keto enzymes involved in protective physiological detoxification of toxic aldehydes formed within cells.

## 5. Conclusions

**Figure 8:**
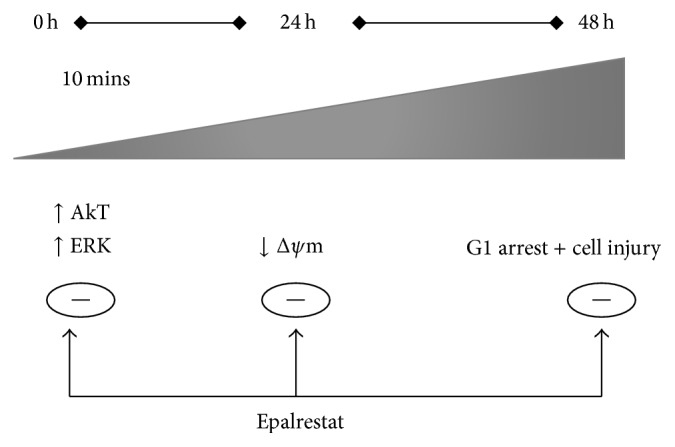
Diagram summarizing the deleterious effects of high glucose on renal tubular cells and the protective role of aldose reductase inhibition against high glucose-induced renal cell injury. Exposure to high glucose acutely activates Akt and ERK pathways in renal cells and causes mitochondrial membrane depolarization and ultimately results in renal cell injury and arrest of cells at G1 phase of cell cycle. Inhibition of aldose reductase via epalrestat reverses these effects (except for cell cycle arrest) and protects renal cells against high glucose-mediated toxicity. Δ*ψ*m, relative mitochondrial membrane potential.

In summary, in vitro exposure to high glucose acutely activates Akt and ERK pathways and induces pathologic changes such as decreases in cell viability and depolarization of mitochondrial membrane potential in renal tubular cells (Figure 8). These pathologic alterations in signaling pathways and cellular organelles initiated by hyperglycemia could potentially contribute to high glucose-induced renal injury. Most importantly, our studies demonstrate that polyol pathway plays a crucial role in development of renal tubular cell injury in a high glucose milieu, and inhibiting its rate-limiting enzyme aldose reductase (AR) protects renal cells against high glucose-mediated deleterious effects (Figure 8). Further studies in vitro and in vivo are required to ascertain their therapeutic potential of AR inhibitors to prevent and/or treat DN.

## Figures and Tables

**Table 1:** Cell cycle analysis (using Tali image-based cytometer) of NRK-52E cells exposed to 30 mM glucose or osmotic control media with or without epalrestat (1 *μ*M) for 48 h.

Treatment groups	Percentage of cells at different stages of cell cycle		
	G1	S	G2
Vehicle-treated control	51.5 ± 1.2	27.75 ± 5.5	19.25 ± 3.5
Vehicle-treated high glucose	59 ± 2	27.33 ± 4.7	13 ± 5.6
Vehicle-treated osmotic control	71.5 ± 4.5	23 ± 5	4 ± 0.1
Control + 1 *µ*M epalrestat	49.75 ± 3.7	26 ± 5.9	21.25 ± 5.9
High glucose + 1 *µ*M epalrestat	61.5 ± 3.9	23.5 ± 5.2	14.5 ± 3.1
Osmotic control + 1 *µ*M epalrestat	62.67 ± 4.3	22.33 ± 6.3	15.67 ± 3.9

## Abbreviations

4E-BP1: Eukaryotic translation initiation factor 4E-binding protein 1
AGE: Advanced glycation end products
AR: Aldose reductase
ARI: Aldose reductase inhibitors
DMEM: Dulbecco's modified Eagle medium
DN: Diabetic nephropathy
ECM: Extracellular matrix
ER: Endoplasmic reticulum
ERK: Extracellular signal-regulated kinase
FBS: Fetal bovine serum
GBM: Glomerular basement membrane
GFR: Glomerular filtration rate
JC-1: 5,5′,6,6′-Tetrachloro-1,1′,3,3′ tetraethylbenzimidazolylcarbocyanine iodide
MAPK: Mitogen-activated protein kinase
MEK: MAPK/ERK kinase
NRK-52E: Normal rat kidney cells
PKC: Protein kinase C
RAAS: Renin-angiotensin-aldosterone-system
TBM: Tubular basement membrane
TGF-*β*1: Transforming growth factor-beta 1
VEGF: Vascular endothelial growth factor.
