# Corrigendum to “Renoprotective Effects of Aldose Reductase Inhibitor Epalrestat against High Glucose-Induced Cellular Injury”

**DOI:** 10.1155/2019/9406241

**Published:** 2019-08-22

**Authors:** Heba El Gamal, Ali Hussein Eid, Shankar Munusamy

**Affiliations:** ^1^College of Pharmacy, Qatar University, P.O. Box 2713, Doha, Qatar; ^2^Department of Pharmacology and Toxicology, School of Medicine, American University of Beirut, Beirut, Lebanon; ^3^Department of Pharmceutical and Administrative Sciences, College of Pharmacy and Health Sciences, Drake University, Des Moines, IA 50311, USA

Following publication of the article, “Renoprotective Effects of Aldose Reductase Inhibitor Epalrestat against High Glucose-Induced Cellular Injury” [1], the authors were made aware that the effects of epalrestat on the high glucose-mediated modulation of Akt and ERK pathways were not clear due to the experimental setup.

An additional experiment was completed to confirm that the exposure of NRK-52E cells to 30 mM high glucose (HG) caused a prominent reduction in the phosphorylation of Akt (about 60% as compared to the control), which was prevented in cells coincubated with epalrestat (EPS; 1 *μ*M) during high glucose exposure. In addition to the original data shown in Figure 2, this suggests that high glucose conditions acutely activate Akt signalling (within 10 minutes of exposure to high glucose) in renal tubular cells whereas sustained exposure to high glucose decreases Akt activation, which could eventually contribute to tubular cell death.

Additionally, the published article incorrectly states that “AR inhibition exerts a protective effect on kidney cells through attenuation of Akt and ERK-dependent pathways” in Discussion. Please read the corrected statement as follows:

Furthermore, the diminished Akt activity in NRK-52E cells following 48 hours of high glucose exposure (Supplemental [Supplementary-material supplementary-material-1]) corroborates with the loss of cell viability in these cells (Figure 1). Interestingly, cotreatment with epalrestat prevented not only the high glucose-induced loss of cell viability but also the diminution of Akt activity in NRK-52E cells.

## Supplementary Materials

Supplementary Materials Supplemental Figure 1: NRK-52E cells were exposed to high glucose (HG) with and without epalrestat (EPS; 1 *μ*M) for 48 hours and assessed for its effect on Akt pathway. (a) A representative western blot showing the expression of p-Akt and total Akt. (b) Bar graph showing densitometry data of p-Akt expression normalized to total Akt expression.
