# Supplementary material for: Corrigendum to “Renoprotective Effects of Aldose Reductase Inhibitor Epalrestat against High Glucose-Induced Cellular Injury”
Source: Biomed Res Int. 2019 Aug 22;2019:9406241. doi: 10.1155/2019/9406241 (PMC6724432; doi:10.1155/2019/9406241)
Supplement: Supplementary Materials — Supplemental Figure 1: NRK-52E cells were exposed to high glucose (HG) with and without epalrestat (EPS; 1 μM) for 48 hours and assessed for its effect on Akt pathway. (a) A representative western blot showing the expression of p-Akt and total Akt. (b) Bar graph showing densitometry data of p-Akt expression normalized to total Akt expression. [file 9406241.f1.docx]

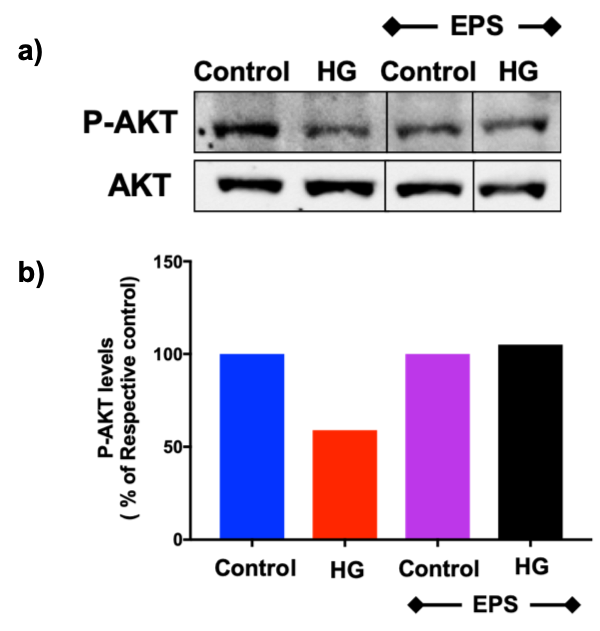


**Supplemental Figure 1**. NRK-52E cells were exposed to high glucose (HG) with and without epalrestat (EPS; 1 μM) for 48 hours and assessed for its effect on Akt pathway. (a) A representative western blot showing the expression of p-Akt and total Akt. (b) Bar graph showing densitometry data of p-Akt expression normalized to total Akt expression.
